# Insights in the Application of Stoichiometric and Non-Stoichiometric Titanium Oxides for the Design of Sensors for the Determination of Gases and VOCs (TiO_2−x_ and Ti_n_O_2n−1_ vs. TiO_2_)

**DOI:** 10.3390/s20236833

**Published:** 2020-11-29

**Authors:** Simonas Ramanavicius, Arunas Ramanavicius

**Affiliations:** 1 Department of Electrochemical Material Science, State Research Institute Center for Physical Sciences and Technology (FTMC), Sauletekio av. 3, LT-10257 Vilnius, Lithuania; simonas.ramanavicius@ftmc.lt; 2 Department of Physical Chemistry, Faculty of Chemistry and Geosciences, Institute of Chemistry, Vilnius University, Naugarduko 24, LT-03225 Vilnius, Lithuania

**Keywords:** titanium dioxide (TiO_2_), non-stoichiometric titanium oxide (TiO_n_ or TiO_2−x_), Magnéli phases (Ti_n_O_2n−1_), gas and volatile organic compound (VOC) sensors, nanomaterials

## Abstract

In this review article, attention is paid towards the formation of various nanostructured stoichiometric titanium dioxide (TiO_2_), non-stoichiometric titanium oxide (TiO_2−x_) and Magnéli phase (Ti_n_O_2n−1_)-based layers, which are suitable for the application in gas and volatile organic compound (VOC) sensors. Some aspects related to variation of sensitivity and selectivity of titanium oxide-based sensors are critically overviewed and discussed. The most promising titanium oxide-based hetero- and nano-structures are outlined. Recent research and many recently available reviews on TiO_2_-based sensors and some TiO_2_ synthesis methods are discussed. Some promising directions for the development of TiO_2_-based sensors, especially those that are capable to operate at relatively low temperatures, are outlined. The applicability of non-stoichiometric titanium oxides in the development of gas and VOC sensors is foreseen and transitions between various titanium oxide states are discussed. The presence of non-stoichiometric titanium oxide and Magnéli phase (Ti_n_O_2n−1_)-based layers in ‘self-heating’ sensors is predicted, and the advantages and limitations of ‘self-heating’ gas and VOC sensors, based on TiO_2_ and TiO_2−x_/TiO_2_ heterostructures, are discussed.

## 1. Introduction

Gas and volatile organic compound (VOC) sensors are important for various safety and environmental control issues. Recently, rather simple gas and VOC sensors based on the measurement of the electrical resistance of the semiconducting layer are developed [1,2,3,4,5,6]. Mostly, semiconducting materials are applied in the design of sensing layer, which is responsible for the formation of analytical signal towards target gases and/or VOCs. Many semiconducting materials, including metal oxides (WO_3_, SnO_2_, Al_2_O_3_, ZnO, TiO_2_, TiO_2−x_ and Ti_n_O_2n−1_) are used as a sensing materials for gas and VOC sensor design. Among many others, metal oxide-based semiconductors [7], many different forms of stoichiometric TiO_2_ and various nonstoichiometric titanium oxides (TiO_n)_ are applied in the design of gas and VOC sensors [8,9], because these titanium-based compounds are rather cheap, nontoxic, biocompatible, chemically stable and insoluble at neutral pHs. Stoichiometric TiO_2_ is n-type semiconducting materials and exist in three main solid phases (anatase, rutile and brookite); therefore, during the formation of sensing layer phase transformations are very often exploited to modify sensitivity and selectivity of formed TiO_2_ layers. [9]. In addition to stoichiometric TiO_2_, recently, various nanostructured non-stoichiometric titanium oxide, which by different authors is abbreviated as TiO_n,_ or TiO_2−x_ and Magnéli phase (Ti_n_O_2n−1_)-based layers, has received significant attention in the development of various sensors for the determination of gaseous materials [8]. These sensors are very sensitive; however, the selectivity of these sensors is still not sufficient, and in addition to that, some of them still have rather high demand of electrical power due to the necessity to power heating element, which almost always is required for the achievement of high temperatures required for the efficient operation of gas and VOC sensitive semiconducting layer. Therefore, some strategies were developed to overcome here mentioned drawbacks: (i) the possibility to apply the ‘self-heating’ mode in some gas and VOC sensors [1,10]; (ii) the engineering of advanced morphology, which offers better selectivity [11,12]; (iii) the formation of core-shell structures [13,14], e.g., quantum dots [15,16], where a core-forming material is covered by shell of particular material, which elicits advanced properties of the resulting hybrid sensing structure [17].

To date, number of reviews on various aspects of TiO_2_ application has been published: (i) comprehensive review on synthesis, properties, modifications, and some applications of TiO_2_-based nanomaterials were provided by Chen and Mao [18]; (ii) Zhang et al. have overviewed environmental and energy applications of titanate- and titania-based nanostructured materials [19]; (iii) principles, mechanisms, and some selected results of photocatalysis on TiO_2_ surfaces were discussed by Linsebigler [20]; (iv) some authors addressed TiO_2_-based nanomaterials [9] and nanoheterostructures [8], which are promoting advanced sensitivity of sensors towards some particular gaseous materials.

The aim of this review is to present insights for the applicability TiO_2−_, TiO_2−x_, Ti_n_O_2n−1_ and TiO_2−x_/TiO_2_-based heterostructures in the design of gas and volatile organic compound (VOC) sensors. We are attracting attention that some of these sensors are able to operate at rather low temperatures and/or are capable of ‘self-heating’, both of which can significantly reduce the consumption of energy required for sensing device.

## 2. Main Structures of Stoichiometric and Non-Stoichiometric Titanium Oxides

**Figure 1:**
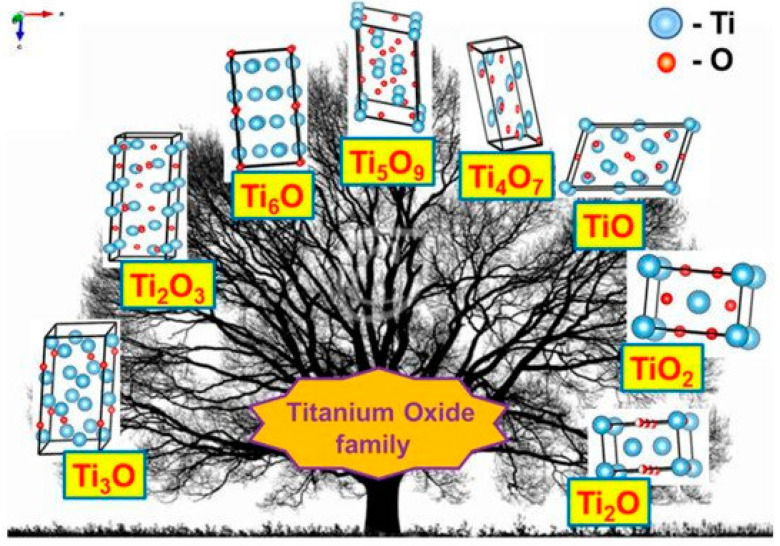
Crystal structures of various titanium oxides. Adapted from [40].

The discovery of the semiconducting properties of TiO_2_ facilitated the application of TiO_2_ in the sensor design. Therefore, various TiO_2_-based structures have been applied in chemical sensors [9,21,22,23,24] and even in biosensors [25,26]. TiO_2_ is a semiconductor of n-type [27] characterized by the charge mobility of 0.4 cm^2^/V s [28]. Three main phases of TiO_2_ have different bandgaps: (i) 3.02 eV for anatase, (ii) 3.23 eV for rutile and (iii) 2.96 eV for brookite [29]. TiO_2_ of all here mentioned phases can be simply formed and easily converted into any here mentioned form by annealing procedure. In addition to those three main phases, titanium oxide is forming various ‘non-stoichiometric’ structures depicted in Table 1. e.g., TiO_n-2_ and/or TiO_n_ and so called Magnéli phases, which have the formula Ti_n_O_2n−1_, where n = 4, …, 10. The most nonstoichiometric structures of titanium oxide (see Figure 1) are exhibiting rather good electrical conductivity and possess sensing properties suitable for the development of gas VOC sensors [1]. Magnéli phases are very often advanced by a ‘close neighbour’ of the Magnéli phase titanium pentoxide (Ti_3_O_5_) with n = 3 for the formula of Ti_n_O_2n−1_. Titanium pentoxide is found in different polymorphic (*α*−, *β*−, *γ*−, *δ*−, and *λ*-forms) [30,31,32,33,34] and it is sometimes mentioned as very first Magnéli phase member, because it has stoichiometric chemical formula, which is consistent with Magnéli phase formula—Ti_n_O_2n−1_ (where n = 3). Ti_3_O_5_ has a monoclinic crystal cell structure (with lattice constants of a = 9.9701 Å, b = 5.0747 Å, c = 7.1810 Å, β = 109.865°). Ti_3_O_5_ is exhibiting superconductivity at temperatures below 3 K, like some other Magnéli phases, e.g., Magnéli phase with n = 4 (Ti_4_O_7_) which is the ‘most respectful’ member of Magnéli phases [35]. Ti_4_O_7_ and other ‘respectful’ members of Magnéli phases in crystal structure are having TiO_2(rutile)_-based shear planes [36,37], which is not the case for Ti_3_O_5_ [35]. Therefore, in our research of XRD-diffractograms, we observed clear signs of Ti_3_O_5_ and TiO_2(anatase)_ without any signs of TiO_2(rutile)_ [1]. It was reported that such Ti_3_O_5_ form of TiO_2−x_/TiO_2_-based heterostructures can be formed at a temperature of 400 °C, which is suitable for the formation of Ti_3_O_5_ with TiO_2(anatase)_-intergrowths [38,39] at very different experimental conditions. The formation of the above-mentioned TiO_2(anatase)_-intergrowths within Ti_3_O_5_ is valuable for the improvement of both electrical and optical sensing properties by the advancement of electrical conductivity and photoluminescence, which are enhanced by formed TiO_2(anatase)_ [1]. However, despite clear advantages, the formation protocols of TiO_2−x_/TiO_2_- and Ti_n_O_2n−1_-based heterostructures are still not well elaborated and most of these structures tend to be oxidized in air atmosphere at higher temperatures. Therefore, such challenges are important to be solved during further research on the development of TiO_2−x_/TiO_2_- and Ti_n_O_2n−1_-based heterostructures and some other materials suitable for the application in gas and VOC sensor design.

## 3. Application of Stoichiometric TiO_2_ in the Design of Gas and VOC Sensors

Gas and VOC sensors based on TiO_2_ are cheap, easily developed. In these sensors rather basic analytical signal registration and assessment methods have been applied for the gas- and VOC-detection procedures. Some TiO_2_-based sensors have rather good sensitivity towards various gaseous materials, which due to their different sensing mechanisms are divided into two major types: (i) reductive gaseous materials—H_2_S, H_2_, CO, NH_3_, CH_3_OH, C_2_H_5_OH, some other gasses and VOCs, (ii) oxidative gases—O_2_, NO_2_ and CO_2_ [20,41,42]. It should be noted that resistivity- or current-based responses of TiO_2_-sensors towards reductive or oxidative gaseous materials are opposite and, due to rather low selectivity of sensing layers’ analysis of gas and VOC mixtures, is still very complicated. Among many different options to measure analytical signal by semiconductor-based gas and VOC sensors, one of the simplest methods is to measure electrical resistivity of the layer. Here, it should be noted that in order to increase reliability of gas and VOC sensors in the analysis of gaseous compounds mixtures, in addition to basic resistivity measurements, it is reasonable to add some additional physicochemical methods, such as an evaluation of photoluminescence signals [1,2,7], which can be applied due to remarkable optical properties of a TiO_2_ semiconductor [43]. It is worth noting that various TiO_2_–based structures can be designed and enhanced by laser pulses, which improve the photocatalytic and photovoltaic performance of designed nano-structures [44].

## 4. Development of Heterostructures Based on TiO_2_ ‘Hybridized’ with Other Semiconductors and Application of Such Structures for the Design of Gas and VOC Sensors

**Figure 2:**
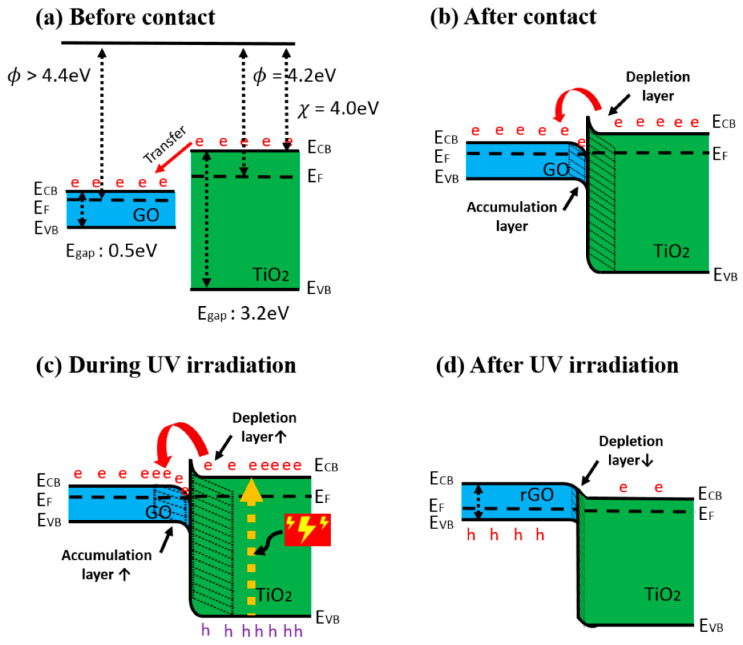
Band diagram of GO/TiO_2_ composite (**a**) before contact, (**b**) after contact, (**c**) during UV irradiation, and (**d**) after UV irradiation. (e: electron, h: hole), adapted from [51].

Heterostructures based on TiO_2_ are rather often applied in the design of gas and VOC sensors. Among them, many different TiO_2_-based heterostructures were investigated: (i) V_2_O_5_/TiO_2_ for chemi-resistive ozone sensors [45], TiO_2_/perovskite heterojunctions for CO gas [46], (ii) TiO_2_/(graphene-carbon)-based toluene VOC sensor operating at room temperature [47], (iii) TiO_2_/SnO_2_ for CO and NO_2_ [48,49], (iv) TiO_2−x_/TiO_2_-structure-based ‘self-heated’ sensor for various reducing gases and VOCs [1]. Improved gas-sensing performance of TiO_2_ by graphene oxide (GO) was demonstrated by Lee et al. [50] (Figure 2a,b), this effect was achieved by UV radiation. The authors claimed that n-n junction had been formed and proposed the band diagram of the GO/TiO_2_, which represent an energetic levels at the hetero-junction. The authors also stated that in such a hetero-junction, the work function of GO was around 4.7 eV [51], which is higher than 4.4 eV that is more usual for GO [52]. An accumulation and depletion layers were formed in this heterostructure [53] (Figure 2b), where, due to the establishment of the Schottky barrier, the depletion layer of TiO_2_ became thicker, and for this reason, the electron number in GO interface increased. The main influence to the change of electrical resistance of this TiO_2_/GO structure was induced by the interaction of adsorbed VOC-molecules on oxygen-based GO functional groups. Moreover, negatively charged adsorbed oxygen supports the catalytic activity of TiO_2_ by lowering the activation energy [54]. TiO_2_ contributed to this heterostructure by its increased gas-adsorption sites and prevention of GO from agglomeration [55,56]. Due to above-mentioned properties, such a structure is sensitive to many reducing gasses, including ammonia gas. However, the active sites of GO were extremely sensitive to moisture, which increased resistance by the interaction of oxygen-based functional groups, such as hydroxyl, carboxylic and carboxyl [57]. This problem was solved by applying UV irradiation, which enabled the increase of both (i) the depletion layer of TiO_2_ and (ii) the accumulation layer of GO (Figure 2c). Therefore, electrons were attracted to GO, where there are reduced oxygen-based groups, and in this way, moisture was removed [58]. The reduced GO was formed, with TiO_2_, a p-n junction, which had a decreased junction width at the interface (Figure 2d). Moreover, photolysis of TiO_2_ led to the injection of electrons into GO, which prevented the recombination of electron-hole pairs [59]. Moreover, the amount of adsorbed oxygen decreased, which also reduced adsorption of water [60]. In one reference [51] it is reported that the number of oxygen-based GO groups was partly restored by photocatalytic action of TiO_2_, and these groups were suitable for the adsorption of reducing VOCs and remained sensitive for over the month.

TiO_2_/SnO_2_-based heterostructures also seem very attractive, due to the peculiarities of the energetic layers of molecular orbitals in TiO_2_, the depletion of electrons is observed when it makes contact with SnO_2_ [49]. Grains of some semiconductors and the most commonly used quantum dots, which are applied in forming the gas- and/or VOC-sensitive layer, are in the range of the Exciton Bohr radius; therefore, they have suitable physicochemical characteristics for the development of various gas and VOC sensors [16]. Therefore, advanced sensing properties can be established by small-grain-based structures [17]. SnO_2_ is characterized by high charge-carrier mobility, which is critically important for the resistivity measurement-based gas and VOC sensors [50,61]. In addition, SnO_2_ is chemically stabile, cheap and forms gas- and VOC-sensing layers, therefore, even in pristine form it is used in various gas and VOC sensors [62]. However, gas- and VOC-sensing temperatures of pristine forms of TiO_2_ and SnO_2_ are rather high, e.g., for pristine SnO_2_ QDs, it is 225 °C [16], but for some SnO_2_-based core-shell structures (e.g., Au/SnO_2_ [63]), these temperatures can be decreased down to 25–80 °C, which makes such structures attractive for real applications because enables to save considerable amount of energy. Therefore, there are some expectations that the formation of SnO_2_ and TiO_2_-based heterostructures will reduce the working temperature of gas and VOC sensors. In the formation of TiO_2_/SnO_2_ heterostructures dedicated for gas and VOC sensors, a variety of different nano-structures were developed: SnO_2_-coated TiO_2_ nanobelts [64], core-shell TiO_2_/SnO_2_ nanofibers [65], TiO_2_-doped SnO_2_ thick films [66], thin films based on layered SnO_2_/TiO_2_ structures [67] and SnO_2_ nanoparticles covered by TiO_2_ nanofibers [49]; SnO_2_ QDs deposited a thin layer of TiO_2_ [68]. In the previously mentioned research, both pristine and modified SnO_2_ QDs were tested in gas and VOC sensors operating in two different modes: (i) at external heating (ii) and at ‘self-heating’ mode, while applying voltages in the range of 1–20 V. Optimal voltage was 20 V and these sensors were sensitive towards NO_2_ and CO. The atomic layer deposition (ALD) method was applied to form TiO_2_-based layers of precise thickness and the authors showed that the selectivity towards both gases and VOCs can tuned by the variation of TiO_2_ layer thickness, the best performance towards CO was yielded when the thickness of TiO_2_ layer was of 30 nm. Moreover, anodic one-dimensional TiO_2_ nanotube provided a large surface area and unidirectional electron transport pathway as a platform for accommodation of thin SnO_2_ coating, which was sensitive towards NO_2_ [69].

Such a finding opens an avenue to tuning the selectivity of sensors based on TiO_2_ layer-modified QDs by rather simple ‘morphology engineering’ procedures.

## 5. Strategies to Reduce Energy Consumption in Gas and VOC Sensors

One strategy to overcome high energy consumption is based on the reduction of the sensing element dimension, another one is based on the application of ‘low temperature’ sensors, which are operating at the temperature of environment where target gases and VOCs are determined, while one additional recently emerging strategy relies on the possibility to apply so called ‘self-heating’ mode in some gas and VOC sensors [1,10]. When the sensing layer has relatively low resistance, if sufficient voltage is applied to the sensing layer and the continuous transfer of charges along this layer is induced by this voltage, then some kinetic energy of these moving charges is converted into the heat due to interaction with various structural elements of the layer. Therefore, during this process, generated heat can be exploited for the increase of sensing layer temperature. ‘Self-heating’ is beneficial in the application of TiO_2−x_/TiO_2_-based structures, because only at a temperature range of 72–180 °C sufficient sensitivity of TiO_2−x_/TiO_2_-based heterostructure towards reducing gases and VOCs has been observed. Such a ‘self-heating’ strategy reduces power consumption, increases the sensitivity of sensor and minimizes the adsorption of water vapour, which is mostly present as a moisture in the environment of interest, and at low temperatures may significantly decrease the sensitivity and selectivity of sensors towards gases and VOCs. Despite of this fact, some researchers are searching for gas- and VOC-sensing materials, which are sensitive towards gases and VOCs at low temperatures [70].

Stoichiometric TiO_2_-based structures have rather high band-gap and, for this reason, they are not well conducting; therefore, it is not always easy to measure the electrical resistance of such layers and even elevation of temperature also does not always sufficiently increase the conductivity up to the suitable conductivity region. ‘Self-heating’ is also not possible if conductivity is low, because in order to get sufficient power, which will heat up the sensor until it reaches a suitable temperature, very high voltages should be applied to the TiO_2_-based structures.

Due to the suitable combination of chemical and semiconducting properties, TiO_2_ has catalytic activity and even photocatalytic activity, both of which can be expressed by the ‘water splitting ability’ or ‘oxidational activity’; these phenomena significantly strengthen when the sensing TiO_2_ structure is irradiated by UV light [44,71]. It was demonstrated that the pulsed laser modification can be successfully employed as a large-scale method to enhance the electronic properties of TiO_2_ nanotubes and such a structure can contribute to the improved photocatalytic or photovoltaic performance of these TiO_2_ nanotubes [72]. Therefore, the additional application of catalytic activity in TiO_2_-based gas and VOC sensors is foreseen; however, it is still rarely exploited, due to significant complexity in operation of such structures. TiO_2_ has been used as a sensing material in room-temperature sensors for formaldehyde [72,73], ozone [45], O_2_ [68], CO [46], C_7_H_8_ [47], ethanol [65,67,74], H_2_ [75,76], and some other gases and VOCs [77].

However, at room temperature (25 °C), the operating sensors are sensitive towards humidity, which is present in the atmosphere. It should be noted that the analytical signals towards humidity and target-gases or VOCs can be very different, e.g., in the presence of humidity (water), conductivity of sensing layer increases, while the presence of the VOC conductivity of the sensing layer has decreased. Such a difference is observed due to different conductance mechanisms realized in the presence of water and the VOCs evaluated here. Water and VOCs adsorbed on the surface of the heterostructure tends to fill the ‘gaps’ between the TiO_2−x_ and/or TiO_2_ grains and in such a way that it increases the conductivity of heterostructures, while the VOCs mostly have significantly lower conductivity compared with gas molecules that are replaced. In order to decrease sensitivity towards humidity, in most cases, the temperature of the sensing layer should be elevated over a room temperature, because the humidity at higher temperatures does not condense well on the sensing structure_-_ A very interesting UV irradiation-based approach to reduce the influence of humidity was demonstrated by Lee et al. [51] and it is discussed in previous chapter. The differing sensitivity towards various gases and VOCs at different temperatures enables the design of sensor-arrays, consisting of similar TiO_2−x_/TiO_2_-based heterostructures, where the individual sensor will operate at the most reliable temperatures, and hence will provide individual signals towards the same mixture of gasses and VOCs [1]. The ‘finger print’ of analytical signals generated by the array can be assessed by ANOVA-based approaches and concentrations of individual gases can be decoded.

## 6. Sensing Mechanism of Some TiO_2_-Based Heterostructures

**Figure 3:**
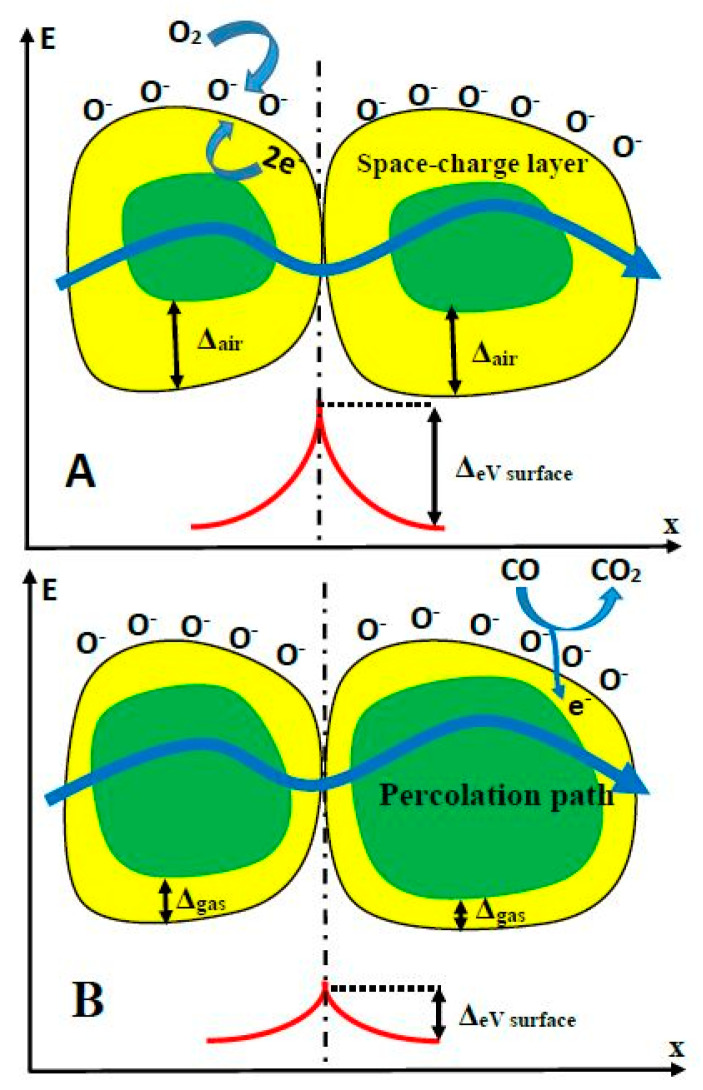
Structural and band models of conductive mechanism upon exposure to CO gas. (**A)** in the absence of CO, (**B)** in the presence of CO.

The sensing mechanism of TiO_2_-sensors is rather complex and it is based on superposition of several processes, such as: (i) target-gas or -VOC adsorption to TiO_2_-surface and (ii) desorption of pre-adsorbed gas or VOC from TiO_2_-surface. These adsorption/desorption processes depend on the nature of gases and lead to the formation of chemical bounds between adsorbed gas, and then the enrichment/depletion of TiO_2_ upper layer by electrons, which are capable of being involved in the charge transfer process and, in such a way, are changing electrical conductivity of TiO_2_-based layer. The conductivity of TiO_2_-layer is based on two main variations: intrinsic conductivity of TiO_2_-grains and the transfer of charge through the boundaries between separated grains. Therefore, both the (i) ratio of grain surface/volume and (ii) the number of such boundaries are very important for the development of the resistivity measurement-based analytical signal. The mechanism of conductance variation in the presence and absence of target gases (e.g., CO) has been discussed in detail by several research groups [78,79] (Figure 3.). The electrostatic interaction between the particular parts of the target analyte and TiO_2_ surface, as well as with the surface of other similarly behaving semiconductors (e.g., WO_3_ [2,7]), also play a significant role in the development of both (i) changes of resistivity [1,2] and (ii) variations of photoluminescence spectra [7,25], which can both be applied for the assessment of the analytical signal [1]. The ‘Debay radius/length’ and the size of grains and are the factors that are significantly affecting charge-transfer efficiency in TiO_2_- and TiO_2−x_-based layers. The TiO_2−x_/TiO_2_-based heterostructure was a highly porous and contained embedded ‘nano-plates’ and ‘nano-sponges’, which enhanced significantly ratio between surface area and volume. Such advanced surface-formations are very useful, because a significantly larger area is available for the target gas or VOC absorption and, in this way, the sensitivity of the sensing layer is enhanced. The above-mentioned factors should be controlled during the development of gas and/or VOC sensors based on TiO_2_ nanostructured layers [42,80,81,82,83,84].

Sorption/desorption of gaseous materials on/from TiO_2_-surface is also a rather complex process and is mainly based on electrostatic and Van der Waals interactions (sometimes called ‘physical-adsorption/desorption’), without the formation/braking of chemical bounds and ‘chemisorption’, which is based on stronger chemical bound formation [85]. The physical sorption is mainly based on electrostatic and Van der Waals interactions between the adsorbed gas or VOC molecules and the TiO_2_ surface. Gases (e.g.,: oxygen), which are present in the environment before the appearance of target gasses, play a critical role in the sensing mechanism, because even before the measurement, they are pre-adsorbed on the surface of the semiconducting layer and, therefore, they affect the initial conductivity of the semiconducting layer. Later, during the course of measurement, these gas molecules are replaced by target (analyte) gas molecules and/or by some interfering gas molecules, when these are present in environment, which is under investigation. In both cases, the adsorbed/desorbed molecules are increasing/decreasing the electrical conductivity and photoluminescence efficiency and some other spectral properties of the TiO_2_-based layer, dependently on their abilities to donate/accept electrons to TiO_2_-based structures and/or electrostatically interact with defect sites, which in the TiO_2_ structure, are responsible for the charge transfer and emission of photoluminescence photons [25]. Both these changes can be interpreted as analytical signals. However, bare TiO_2_-based gas and VOC sensors have rather high electrical resistance and low dynamic range, which determine rather low sensitivity and selectivity. Therefore, heterostructures based on TiO_2_ and other metal oxides, carbides and other materials have been designed [82,83,86,87,88,89]. Conducting polymers (such as polypyrrole (Ppy) and polyaniline (PANI)) can be applied in the formation and modification of TiO_2_-based heterostructures sensitive to gasses. The TiO_2_/Ppy-based sensing heterostructures are able to operate at rather low temperatures. The TiO_2_/Ppy-based sensor is suitable for the detection of gaseous NH_3_ [90,91] and the main LPG components (propane and butane) [92]. The TiO_2_/PANI-based sensors for the determination of NH_3_ has been reported [92,93,94,95]. The advanced sensitivity of TiO_2_/Ppy and TiO_2_/PANI-based sensors is based on the establishment of n/p-junction at TiO_2_/Ppy interphase.

## 7. Recent Achievements and Perspectives in the Application of Non-Stoichiometric Titanium Oxides for Gas and VOC Sensor Design

The conductivity of TiO_2−x_ is considerably higher than that of stoichiometric TiO_2_. Especially well this effect is observed for Ti_n_O_2n−1_ when ‘n’ is between 4 and 10, because at this stoichiometry, Magnéli phases can be formed rather easily [96]. These phases possess metallic conductivity, super-conductivity and some other valuable properties [97,98], which can be applied in the design of gas and VOC sensor. It was determined that Magnéli phases are forming in TiO_2−x_-based layers [99] planes based on Ti_n_O_2n−1_ moieties, which penetrate through a matrix of TiO_2_, and along this shear-plane, rather good conducting zones with electron-transfer-based conductivity are observed [98]. Even the presence of not-real members of Magnéli phases (Ti_2_O_3_ and/or Ti_3_O_5_) significantly increases the conductivity of titanium oxide-based heterostructures [1,35]. Such conductivity variations are well exploited in the memristor-type logical elements, where the electrical resistivity of TiO_2_ is increased and/or decreased by the reduction and/or oxidation of TiO_2_-based structure when the corresponding potentials are applied [100].

The Ti^3+^-containing TiO_2−x_/TiO_2_-based heterostructure possess some ‘oxygen vacancies’ that are responsible for the electron mobility in this n-type semiconducting structure [101]. In our earlier research, we predicted that such ‘oxygen vacancies’ provide increased sensitivity towards some reducing and oxidizing gases and VOCs [1]. TiO_2_-based structures can be reduced and form non-stoichiometric titanium oxides (TiO_2−x_) and even Ti_n_O_2n−1_-based Magnéli phases [97]. In the TiO_2−x_ structure characterized by low ‘x’ value, which varies in the range of 0 < x < 0.10, ‘point defects’ dominate in the crystal structure, which possess great number of ‘oxygen vacancies’ and interstitials based on Ti^3+^ and Ti^4+^ [102]. The number of defects increases together with the increased ‘oxygen deficiency’ rate. In some research, it is reported that, in Magnéli phases with an ‘x’ value of 0.10 < x < 0.34, crystallographic shear planes and planar defects are extended [99,103]. Magnéli phase-based titanium oxide structures conduct well and are chemically stabile; therefore, they have found many application areas in the development of fuel cells, batteries and waste water decontamination [104,105,106]. The same advantageous properties are suitable for the development of gas and VOC sensors. Hence, the presence of TiO_2−x_/TiO_2_-based heterostructures in TiO_2_-based sensors can improve the sensitivity of semiconducting layer, due to the advanced conductivity.

**Figure 4:**
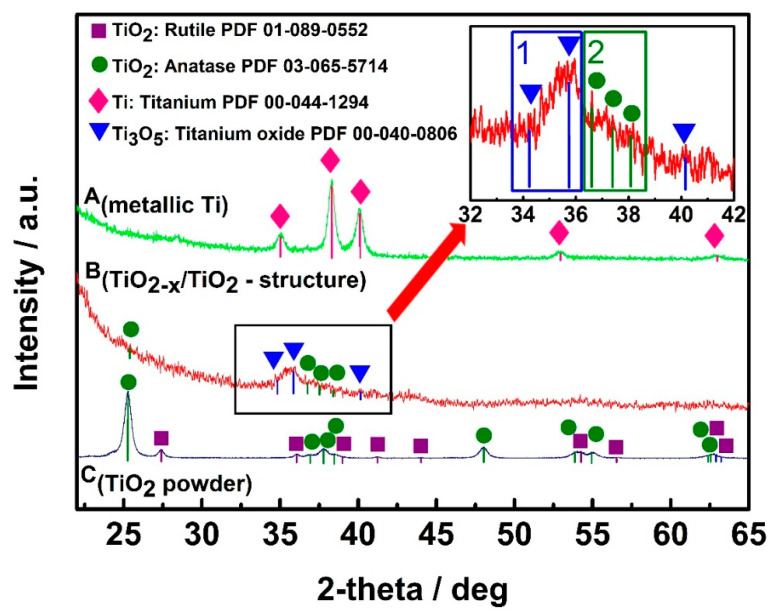
XRD patterns of: **A_(metallic Ti)_**—metallic Ti layer of 100 nm thickness, which was formed by magnetron sputtering; **B_(TiO2−x/TiO2-structure)_**—TiO_2−x_/TiO_2_(400 °C)-based heterostructure, which was formed from above mentioned metallic 100 nm thick Ti layer; **C_(TiO2 powder)_**—TiO_2_-powder, which was used as ‘control sample’ and by supplier (Sigma-Aldrich) was declared as 99.3% TiO_2_ in anatase phase. Figure adapted from [1].

The design and technical procedures applied during the creation of TiO_2_-based sensors at high extent determines sensitivity and selectivity of developed gas and VOC sensors. Non-conducting substrates with formed interdigitated electrodes are mostly used for the deposition of TiO_2_- and TiO_n_- and TiO_2−x_-based sensing structures. TiO_2_/TiO_2−x_ TiO_2−x_/TiO_2_ heterostructure can be formed by hydrothermal oxidation (in aqueous alkaline solution) of metallic titanium layer, which should be pre-deposited by magnetron sputtering. Therefore, additional strategies for the increase of conductivity should be elaborated, and one of such strategies is the modification of stoichiometric TiO_2_-based structures by some non-stoichiometric TiO_2_ [1]. TiO_2−x_/TiO_2_-based structures, which, due to the formation of Ti^3+^, has some Ti_n_O_2n−1_ clusters characterized by significantly advanced electrical conductivity, can be formed using many different approaches, e.g., metallic zinc-based reduction [107], plasma treatment [108], laser irradiation [109], high-energy particle bombardment [110] and thermo-chemical treatment [111]. The growing of large Ti_3_O_5_ crystals is still very challenging due to polymorphism of titanium oxides [35]. XRD diffractograms (Figure 4) of designed TiO_2−x_/TiO_2_-based heterostructures, represents well some dispersion of Ti-based oxides [1], which form the TiO_2−x_/TiO_2_-structure, but the other research [112] confirms the broad ‘XRD peak-reach area’ between 27° and 37° in the XRD-diffractogram [1] is assigned to Ti_3_O_5_, Ti_4_O_7_ and/or Ti_8_O_15_, which reveals the formation of the Magnéli phases within the TiO_2−x_/TiO_2_-based heterostructure. Meanwhile, the XRD peak between 34° and 37° reveals the presence of γ-Ti_3_O_5_, similar peaks were present in XRD-PDF file ‘00-040-0806 for *γ*-Ti_3_O_5_′ and by some other researchers [35], where the γ-Ti_3_O_5_ was formed by pulsed laser deposition. Low-temperature superconductivity was reported for both Ti_4_O_7_ and γ-Ti_3_O_5_ layers [35]; moreover, this research reports rather broad peaks in XRD-difractograms of γ-Ti_3_O_5_ and of Ti_4_O_7_ at 36–38° and at 42–43°, respectively_._ XRD peaks and some other shape-like-features in the same area of XRD-difractograms at very similar signal to noise ratio were also registered in the research, which is dedicated for sensing application of TiO_2−x_/TiO_2_-based heterostructures [1]. It should be noted that here commented XRD-diffractograms of TiO_2−x_-based layers [1,35,113] despite of the here-mentioned very distinct similarities in all XR-difractograms have also some peculiarities, which appeared due to the different preparation procedures of TiO_2−x_-based layers during each of the here-mentioned research. This fact reveals that there is a lot of space to change/vary the intrinsic morphology and composition of TiO_2−x_/TiO_2_-based heterostructures and to tune selectivity a sensitivity of such heterostructures by the establishment of different TiO_2−x_/TiO_2_-stoichiometry that is achieved by different annealing temperatures and/or durations [1].

It should be noted that a significant part of the here-covered research on Magnéli phases has been based on the evaluation of powders and little research was performed on other structures, such as fibres [101] or planar structures [1]. In our research, we formed TiO_n_-based ‘Magnéli phase like structures’ using the hydrothermal oxidation of thin metallic layer of titanium sputtered by magnetron deposited [1]. Differently from above-mentioned research, in some particular cases, the ratio of non-stoichiometric titanium oxides vs. stoichiometric titanium oxides in TiO_2−x_/TiO_2_-based heterostructures and/or in TiO_2_-based layers can be increased by the treatment based on heating at a high temperature in the presence of reducing gasses and VOCs [113]. Some studies were dedicated for the investigations of transition between metal and insulator states of Ti_3_O_5_ that were performed: (i) applying visible-light pulses that have induced transition between β and λ forms of Ti_3_O_5_ [33], (ii) thermal treatment induces transition between α and β forms at 450 K [30] and between δ and γ at 240 K [31,32,33,34]. The transition between metal and insulator states at 350 K was observed [35]. Various research illustrates that temperature range between 240 and 450 K is important for the phase changes of Ti_3_O_5_ and is important for the tuning of titanium oxide layer conductivity and the adaptation of this layer for the determination of gaseous compounds. Hence, TiO_2_ can be turned into Ti_n_O_2n−1_ by proper doping, reduction and/or partial oxidation of metallic titanium [1] and later particular temperature-based treatment enables the establishment of the optimal TiO_2−x_/TiO_2_-based heterostructures. Therefore, such structures after some further adjustments can be applied in the design of sensors, which will have different levels of sensitivity and selectivity. Therefore, such sensors with different levels of selectivity will be suitable for the establishment of sensor arrays. However, despite their advantageous conductivity, catalytic activity and sensing performance, which are all very suitable for the development of gas and VOC sensors, non-stoichiometric titanium oxides have a significant disadvantage in comparison to stoichiometric ones, which is based on lower stability at the atmospheric condition, due to the continuous oxidation into stoichiometric TiO_2_. Moreover, the selectivity of all the above-mentioned sensors is still rather low. Therefore, significantly better understanding of the formed structures and the correlation of the here-mentioned procedures with the sensing properties of the formed semiconducting TiO_2−x_/TiO_2_-based heterostructures, which will meet all the specific requirements of the particular applications, is still required.

## 8. Analytical Signal Registration Protocols

The selection of a suitable method for the registration of the analytical signal is an important issue in the design of gas and VOC sensors; therefore, for this purpose, various methods can be applied: most of them are based on galvanostatic, potentiostatic and variety of potentiodynamic measurements. Some of these electrical signal registration methods can, at the same time, be useful for the ‘self-heating’ of sensing structure and/or, if well-adjusted, are suitable for the increase of sensitivity and selectivity of sensors. Electrical conductivity measurements are the most frequently applied for the determination of the analytical signal by sensors based on all types of stoichiometric and non-stoichiometric titanium oxides [1,3,4,6,9,12,14,15,16,23,24,41,67,74,77,81,86,90,91,94]. The conductivity of TiO_2−x_/TiO_2_-based gas- and/or VOC-sensitive structure is rather high in comparison to that of stoichiometric TiO_2_, therefore, current passing through TiO_2−x_/TiO_2_-based structure heats-up sensing layer, which is very useful for the achievement of different selectivity at different temperatures, while the analytical signal can be based on changes of current registered at the same constant potential, which is applied for the heating of system and/or the evaluation of photoluminescence spectra [1]. However, it should be noted that stoichiometric titanium oxide-based sensors are characterized by rather low electrical conductivity (~10^−10^ S/m); therefore, the significant elevation of temperature up to 200–400 °C is required to increase the conductivity, in order to reach optimal sensing conditions. This requirement is typical for most of the sensors based on stoichiometric TiO_2_-based semiconducting materials; therefore, in most of the stoichiometric TiO_2_-based sensors, significantly higher temperatures should be applied in comparison to optimal temperatures for nonstoichiometric TiO_2_-based sensors [1]. Operation at higher temperatures increases the energy consumption of sensor, which becomes very actual if the sensors are operating at autonomous powering.

Despite of its very good sensitivity, the selectivity of all types of titanium oxide based sensors is still rather poor. Therefore, additional signal registration methods are very beneficial, especially if they are applied simultaneously with electrical resistance measurements. The application of photoluminescence detection-based analytical methods in the development of TiO_2_- and TiO_2−x_/TiO_2_-based gas and VOC sensors can significantly advance analytical information, which can be gathered by electrochemical methods. Most of the stoichiometric and nonstoichiometric titanium oxide structures are characterized by photoluminescence spectra with a maximum in the range between *λ* = 415–500 nm. Differences of photoluminescence spectra of titanium oxide structures depends on annealing conditions (manly on the duration of heating and temperature changing protocol), both of which are very important during the formation of the structures. Dependently of these conditions, different phases of stoichiometric and/or nonstoichiometric titanium oxide are formed, e.g., when TiO_2−x_/TiO_2_-based structure is formed using annealing at 400 °C, then significantly more intense photoluminescence signal (higher about 10 times) has been created in comparison to that determined for the TiO_2−x_/TiO_2_-based heterostructure formed using annealing at 600 °C, and if TiO_2−x_/TiO_2_-based structure is formed by annealing at 800 °C the photoluminescence of such a structure is very weak—over hundred times lower than that of TiO_2−x_/TiO_2_ -based heterostructure prepared at 400 °C [1]. Therefore, during the design of stoichiometric and nonstoichiometric titanium oxide-based structures for optoelectronics-based sensors preparation conditions should be followed and photoluminescence and sensing properties of these structures can be rather easily tuned by the adapting proper annealing conditions.

The increase of heating voltage, which is simultaneously used for the measurement of conductivity of the TiO_2−x_/TiO_2_-based heterostructure, gradually decreases photoluminescence peak intensity [1], due to the temperature dependent reduction of number of photoluminescence emitting centers at higher temperatures and temperature-based photoluminescence quenching [106,114,115]. In addition, the shift of peak maximum (*λ*_max_), which is usually determined as a maximum of Gauss function, towards shorter wavelengths was observed [1]; this fact reveals that photoluminescence emitting centers, which are located close to the surface of TiO_2−x_/TiO_2_-based heterostructure are electrostatically affected by adsorbed gaseous material [25]. The evaluation of TiO_2(anatase)_ photoluminescence was evaluated in some research and it was determined that photoluminescence is excited at shallow trap levels that have energy levels in the range of 0.41–0.64 eV, which is below the conduction band of this oxide [116]. Narrow photoluminescence emission bands were determined for TiO_2(anatase)_ powder; they were emitted by self-trapped excitons that are appearing in crystal structure of TiO_2(anatase)_, which is based on octahedral sheets of ‘TiO_6_‘ structural elements [19,117]. Therefore, electrical resistivity measurements can be extended by the determination of photoluminescence signal, which can also be applied for target gas and VOC determination by titanium oxide-based sensors. The evaluation of photoluminescence-decay kinetics can be applied for the determination of self-trapped exciton-based origin of photoluminescence, which, among titanium oxides, is the most characteristic for crystalline TiO_2(anatase)_ [118], and during the formation of sensing layer, it can be exploited as a confirmation of TiO_2(anatase)_ presence in the composition of sensing layer [1] and for the determination of analytical signal. In addition to registration of conventional photoluminescence signal, the evaluation of photoluminescence-decay kinetics can provide new options for the evaluation of quality of sensing layer [1,7] and the determination of analytical signal by sensors based on stoichiometric and non-stoichiometric titanium oxides.

It should be noted that the selectivity of gas and VOC sensors still remains not very high, even if very good sensitivity has been achieved in a lot of research [1,3,4,6,9,12,14,15,16,23,24,41,67,74,77,81,86,90,91,94]. Therefore, approaches suitable for the evaluation of gas and/or VOC mixtures should be elaborated. In this context, it is very promising that the variation of sensing layer temperature significantly changes both the selectivity and sensitivity of gas and/or VOC sensors, which was well demonstrated by the gas- and VOC sensor based on TiO_2−x_/TiO_2_-layer [1], and this effect can be applied in the design of sensor arrays, where sensors with different selectivity and sensitivity can provide very different responses in the same gas and VOC mixture and these ‘finger prints’ registered by such a sensor array can be assessed and decoded by applying the corresponding methods of the multi-variation mathematical analysis.

## 9. Conclusions and Future Developments

Despite the significant progress in the development of stoichiometric TiO_2_-based heterostructures, practical application in real sensing devices is still limited. However, recently, some research has demonstrated that hydro-thermal oxidation of metallic titanium or reduction of TiO_2_ layers leads to the formation of heterostructures based on TiO_2−x_/TiO_2_ and/or Ti_n_O_2n−1_ heterostructures. Recent developments turn to the prediction that analytical characteristics of these sensing layers can be tuned by the adjustment of TiO_2−x_/TiO_2_/Ti_n_O_2n−1_ ratio in formed heterostructures. Formed structures can be integrated into gas and VOC sensors as the ‘self-heated’ sensing layer. In the sensor based on this heterostructures, the variation of electrical current, which is flowing through TiO_2−x_/TiO2-based heterostructure, at fixed potential, is registered. ‘Self-heating’ is beneficial for the application of TiO_2−x_/TiO_2_-based structures, because only in the temperature range of 72–180 °C sufficient sensitivity of TiO_2−x_/TiO_2_-based heterostructure towards reducing gases and VOCs has been observed. We expect that titanium oxide-based heterostructures, in the future, will find more advanced applications in gas and VOC sensors. However, a more advanced and comprehensive understanding of how to control the transition between the differently conducting titanium oxide-based layers is still required, in order to achieve desirable semiconducting properties, which are the most optimal for applications in sensors for the determination of gases and VOCs. Therefore, by the elaboration of the successful sensing layer formation and signal registration protocols, the advantageous properties of nonstoichiometric titanium oxide (TiO_2−x_ and/or Ti_n_O_2n−1_)-based structures can be well applied in the design of gas and VOCs sensors.

## Figures and Tables

**Table 1:** Variation of the crystal structure of titanium oxides with O/Ti stoichiometry. Adapted from [40].

Compound	X in TiO_x_	Structure
TiO_2_	2	Rutile
Ti_10_O_19_	1.9	Anatase
Ti_9_O_17_	1.89	Triclinic
Ti_8_O_15_	1.875	Triclinic
Ti_7_O_13_	1.857	Triclinic
Ti_6_O_11_	1.833	Triclinic
Ti_5_O_9_	1.8	Triclinic
Ti_4_O_7_	1.75	Triclinic
γTi_3_O_5_	1.67	Monoclinic
Ti_2_O_3_	1.5	Tetragonal
TiO	1	Hexagonal
		Cubic Monoclinic
Ti_2_O	0.5	Hexagonal
Ti	0	Hexagonal
